# In this month’s Bulletin

**DOI:** 10.2471/BLT.16.001116

**Published:** 2016-11-01

In the editorial section, Dermot Maher & Giorgio Cometto (786) explain why research on community-based health workers is needed to achieve the sustainable development goals. Naoko Ishikawa et al. (787) report elimination of mother-to-child transmission of HIV and syphilis in Cuba and Thailand.

In the news section, Vijay Shankar Balakrishnan (790–791) reports on the difficulties transgender people can encounter when accessing health services and interviews Yelzhan Birtanov (792–793) about efforts to modernize the provision of health care in Kazakhstan.

## Australia

### Hepatitis B immunization

Andre Louis Wattiaux et al. (826–834) quantify vaccine-preventable infections in indigenous adults.

## Brazil

### Tracking microcephaly

Juliana Sousa Soares de Araújo et al. (835–840) establish baselines and trends for congenital Zika syndrome.

## Australia, the Netherlands, New Zealand, the United Kingdom of Great Britain and Northern Ireland & the United States of America. 

### Validating disability weights 

Belinda J Gabbe et al. (806–816) use patients’ reports to calculate the long-term effects of injuries. 

## Haiti 

### Concurrent chikungunya, dengue and malaria

Mathieu JP Poirier et al. (817–825) test children for multiple infections.

## Japan

### Surviving a nuclear disaster

Claire Leppold et al. (859–860) explore longer-term public health considerations.

## Global

### Tracking inequalities in vaccination 

María Clara Restrepo-Méndez et al. (794–805) analyze trends of immunization coverage in 86 low- and middle- income countries. 

### Ensuring safe blood supplies

Justin Lessler et al. (841–849) review the evidence for serological milestones in Zika virus infection.

### Arbovirus control dilemmas

Hugo López-Gatell et al. (850–855) discuss the implications of dengue vaccine policies.

### Monitoring health inequality

Ahmad Reza Hosseinpoor & Nicole Bergen (856–858) make a case for using area-based units of analysis.

